# Endocrine complications of anorexia nervosa

**DOI:** 10.1186/s40337-023-00744-9

**Published:** 2023-02-15

**Authors:** Melanie S. Haines

**Affiliations:** 1 Neuroendocrine Unit, Massachusetts General Hospital, 50 Staniford Street, Suite 750B, Boston, MA 02114 USA; 2 Harvard Medical School, Boston, MA USA

**Keywords:** Anorexia nervosa, Endocrine complications, Amenorrhea, Low bone density

## Abstract

An important component in the treatment of anorexia nervosa (AN) is the evaluation and management of its endocrine complications, including functional hypogonadotropic hypogonadism and increased fracture risk. The body’s adaptive response to chronic starvation results in many endocrine abnormalities, most of which are reversible upon weight restoration. A multidisciplinary team with experience in treating patients with AN is critical to improving endocrine outcomes in patients with this disorder, including in women with AN who are interested in fertility. Much less is understood about endocrine abnormalities in men, as well as sexual and gender minorities, with AN. In this article, we review the pathophysiology and evidence-based recommendations for the treatment of endocrine complications in AN, as well as discuss the status of clinical research in this area.

## Plain English summary

Endocrine complications are common in anorexia nervosa (AN). Examples include absence of menstrual periods in women, low testosterone levels in men, and low thyroid hormone levels, all of which are considered adaptive to the state of undernutrition. Low bone density and increased risk of fractures are also common complications. In women, weight recovery and restoration of menstrual periods are important treatments for low bone density. Less is known about the treatment of endocrine complications in men with AN. In this article, we review the causes and treatment of endocrine abnormalities in AN.

## Introduction

A critical component of the treatment of anorexia nervosa (AN) is the evaluation and management of its medical complications that affect nearly every organ system and contribute to it having one of the highest mortality rates among psychiatric disorders [1]. The body’s adaptive response to chronic starvation results in many endocrine abnormalities, most of which are reversible upon weight restoration, but some abnormalities may persist despite treatment of the underlying disease. Fewer than half of patients with AN fully recover from the disorder, one-third improve but only partially recover, and one-fifth remain chronically ill with anorexia nervosa [1], which makes endocrine complications an important consideration in the long-term management of the disorder. In this review, we will discuss the pathophysiology and clinical implications of the effects of AN on hypothalamic-anterior pituitary hormone axes (i.e., gonadal axis, adrenal axis, thyroid axis, and the growth hormone/insulin-like growth factor 1 axis); hypothalamic hormones secreted by the posterior pituitary (i.e., oxytocin and anti-diuretic hormone); gut-derived appetite regulating hormones (e.g., ghrelin and peptide YY); body composition, adipokines, and myokines; bone marrow adipose tissue and skeletal integrity; and fertility and pregnancy outcomes. Table 1 provides a summary of the hormone abnormalities in AN that are discussed in this review.

**Table 1:** Hormone abnormalities in anorexia nervosa (AN)

Hormone	Source	Relevant function in AN	Levels in AN
Hypothalamic-pituitary–gonadal axis			
Gonadotropin-releasing hormone (GnRH)	Hypothalamus	Stimulates LH and FSH pulsatility	↓
Luteinizing hormone (LH)	Anterior pituitary	Stimulates ovulation (women), stimulates testosterone production (men)	↓
Follicle stimulating hormone (FSH)	Anterior pituitary	Stimulates folliculogenesis and estradiol production (women), stimulates spermatogenesis (men)	↓
Estradiol	Ovaries (women); adipose tissue (men)	Stimulates osteoblasts and inhibits osteoclasts, ?anxiolytic effects	↓
Testosterone	Testes (men); ovaries (women)	Stimulates osteoblasts and inhibits osteoclasts, ?anti-depressent effects	↓
Hypothalamic–pituitary–adrenal axis			
Corticotropin-releasing hormone (CRH)	Hypothalamus	Stimulates ACTH pulsatility	↑
Adrenocorticotropic hormone (ACTH)	Anterior pituitary	Stimulates cortisol production	↑
Cortisol	Adrenal glands	Inhibits LH and FSH pulsatility, inhibits osteoblasts and stimulates osteoclasts, ?anxiety and depressive effects	↑
Dehydroepiandrosterone sulfate (DHEAS)	Adrenal glands	?Anxiolytic effects	No consensus
Hypothalamic-pituitary-thyroid axis			
Thyroid-releasing hormone (TRH)	Hypothalamus	Stimulates TSH production	↓ ← →
Thyroid stimulating hormone (TSH)	Anterior pituitary	Stimulates T4 and T3 production	↓ ← →
Thyroxine (T4)	Thyroid	Increases metabolism	↓ ← →
Triiodothyronine (T3)	Thyroid	Increases metabolism, ?Anti-depressant effects	↓
Reverse T3 (rT3)	Thyroid	Metabolically inactive	↑
Growth hormone-insulin-like growth factor 1 axis			
Growth hormone (GH)	Anterior pituitary	Stimulates gluconeogenesis and lipolysis, stimulates osteoblasts and inhibits osteoclasts	↑ (GH resistance)
Insulin-like growth factor 1 (IGF-1)	Liver	Stimulates linear bone growth in adolescents with open physes, stimulates osteoblasts and inhibits osteoclasts	↓
Hypothalamic hormones antidiuretic hormone and oxytocin			
Antidiuretic hormone	Hypothalamus	Stimulates water reabsorption in the kidney	↑ ← →
Oxytocin	Hypothalamus	?Anxiolytic and anti-depressant effects, stimulates osteoblasts and inhibits osteoclasts	↓
Gut-derived appetite regulating hormones			
Ghrelin	Gastrointestinal tract (mostly gastric cells)	Orexigenic, inhibits LH and FSH pulsatility, stimulates ACTH and GH secretion	↑
Peptide YY (PYY)	Gastrointestinal tract (endocrine L cells)	Anorexigenic	↑ ← →
Adipokines, myokines, and hepatokines			
Leptin	Adipose tissue	Anorexigenic, stimulates kisspeptin signaling to GnRH neurons	↓
Myostatin	Skeletal muscle	Inhibits muscle growth	↓
Preadipocyte factor 1 (Pref-1)	Adipose tissue	Inhibits osteoblast and adipocyte differentiation	↑
Fibroblast growth factor 21 (FGF-21)	Liver	Inhibits intracellular effects of GH	↑

## Hypothalamic-pituitary–gonadal axis

Although amenorrhea is no longer a diagnostic criterion for AN in the Diagnostic and Statistical Manual of Mental Disorders, 5th edition (DSM-5), 66–84% of women with AN have amenorrhea (defined as absence of menstruation for greater than three months), and 6–11% of women with AN have oligomenorrhea (defined as fewer than nine menstrual cycles over a 12-month period or cycle length greater than 35 days) [2, 3]. In AN, serum levels of leptin (an adipokine) are reduced consequent to low fat mass, which results in decreased kisspeptin signaling to gonadotropin-releasing hormone (GnRH) neurons in the hypothalamus [4]. Reduced GnRH pulsatility results in reduced LH and FSH pulsatility from the anterior pituitary, and consequently reduced estradiol secretion from the ovaries in women and testosterone secretion from the testes in men. This condition is called functional hypogonadotropic hypogonadism. Increased ghrelin and cortisol levels in women with AN may also play a contributory role to functional hypogonadotropic hypogonadism [5, 6]. Before a diagnosis of functional hypogonadotropic hypogonadism/functional hypothalamic amenorrhea can be made in a woman with AN, other causes of amenorrhea must be excluded, including pregnancy, thyroid dysfunction, and premature ovarian insufficiency. Among men, the diagnosis of functional hypogonadotropic hypogonadism relies on ascertaining symptoms and signs consistent with testosterone deficiency, obtaining at least two morning fasting testosterone levels with low or inappropriately normal gonadotropins, and excluding other causes of hypogonadotropic hypogonadism if clinically indicated [7].

Although weight recovery is generally thought to restore GnRH pulsatility, inter-individual differences exist in the sensitivity of the hypothalamic–pituitary–gonadal axis to weight gain (and loss). Weight may need to be restored for 6–12 months for menses to resume. Despite weight recovery, amenorrhea may persist in up to 15% of adolescent girls and women with AN [8, 9]; when to re-evaluate for other causes of amenorrhea should be individualized based on the clinical situation. In such cases, menstrual function may not resume due to continued eating disorder psychopathology or relatively less body fat despite a normal body weight. Among men with AN, levels of serum testosterone generally increase into the normal range with weight gain [10]. Given the paucity of data on the efficacy and safety of testosterone therapy in men with chronic illnesses such as AN, the Endocrine Society does not provide general recommendations for testosterone therapy in patients with chronic illness. Instead, the decision to treat these men must be individualized based on the severity of hypogonadism, comorbid conditions, patient preferences, and the potential benefit vs. risk profile [7].

Hypogonadotropic hypogonadism is hypothesized to contribute to comorbid mood disorders in AN. Administration of transdermal estradiol has been shown to improve anxiety in adolescent girls with AN independent of changes in weight [11]. Since the ovaries make both estrogen and testosterone, hypogonadotropic hypogonadism results in hypoandrogenemia in women with AN, which has been associated with more severe depression and anxiety independent of body weight [12]. Although administration of transdermal testosterone demonstrated antidepressant effects in a three-week study of women with AN and depression [13], testosterone therapy for 24 weeks did not improve depression or anxiety symptoms compared with placebo in women with AN [14].

Although amenorrhea is common among women with AN, large cohort studies have demonstrated that women with AN are at a two-fold greater risk of unplanned pregnancy than women in the general population [15, 16]. This may be because of a lack of understanding among patients, and lack of counseling from providers, that ovulation can occur in the absence of menstruation. The importance of contraception should therefore be discussed with all patients with AN. An unplanned pregnancy increases the likelihood that a woman is unaware of her pregnancy, delays prenatal care, engages in unsafe behaviors, and does not get adequate nutrition, all of which increase the risk to both mother and fetus.

When seeking medically assisted reproduction, the majority of women with a history of an eating disorder do not report it to their providers, and the majority of providers do not screen for a history of an eating disorder [17, 18]. This is a critical unmet need as pregnancies in women with AN, whether planned or unplanned, are at higher risk for adverse outcomes for both mother and fetus, including miscarriage, cesarean delivery, premature birth, low birth weight, and perinatal mortality [19]. Managing AN in pregnancy therefore requires a multidisciplinary team with experience in managing high-risk pregnancies.

## Hypothalamic–pituitary–adrenal axis

Cortisol is a glucocorticoid hormone made by the adrenal glands that modulates the body’s response to stress by regulating metabolism, blood pressure, and blood glucose levels, suppressing inflammation, etc. Hypercortisolemia, including elevated 24-h urine free cortisol [20] and midnight salivary cortisol levels [21] and inappropriate suppression of serum cortisol levels on dexamethasone suppression testing [22], is present in about one-third of patients with AN. The degree of hypercortisolemia is inversely associated with BMI and fat mass [5], and rarely exceeds two times the upper limit of the reference range. Chronic stress and starvation, including elevated ghrelin levels [23], are thought to activate the hypothalamic–pituitary–adrenal axis through increased corticotropin-releasing hormone (CRH) secretion from the hypothalamus and adrenocorticotropic hormone (ACTH) secretion from the anterior pituitary [24]. Some studies have suggested that CRH hypersecretion may also play a role in the pathophysiology of the disorder, as central injections of CRH in animals leads to anorexia and increased motor activity [25], which can be reversed by CRH antagonists [26]. Higher levels of serum cortisol are also positively associated with depression and anxiety measures [27] and eating disorder psychopathology [28] in women with AN, but whether hypercortisolemia is a cause of these psychiatric symptoms, or merely a marker of more severe disease, is unclear. In contrast to the consistent findings of hypercortisolemia in patients with AN, data regarding levels of adrenal androgens, i.e., dehydroepiandrosterone sulfate (DHEAS), in AN are conflicting, with studies demonstrating low, normal, or elevated levels compared with the reference range or controls [29–31].

Among adolescent girls and women with AN, higher cortisol levels predict greater increases in fat mass, and particularly trunk fat mass, during weight recovery [5, 32]. With weight recovery, normalization of cortisol levels is observed in many, but not all, patients with AN [22]. Whether the cause of persistent hypercortisolemia is continued eating disorder psychopathology in some of these women is unclear; consistent with this hypothesis, one study demonstrated that a persistently abnormal 1 mg dexamethasone suppression test predicted a high risk of relapse of AN [33]. Pharmacologic approaches to lower cortisol levels in patients with AN are not recommended, especially considering that such therapies could result in weight loss or precipitate adrenal insufficiency. In contrast, DHEA administration for 12 months may improve some psychological parameters in women with AN as demonstrated in one study [34], but further studies of DHEA with long-term safety data are required. For instance, oral DHEA and estrogen replacement may be detrimental to bone in adolescent girls with AN and open epiphyses [35].

## Hypothalamic-pituitary-thyroid axis

Thyroid hormones (thyroxine (T4) and triiodothyronine (T3)) are important regulators of metabolism that affect the function of organs throughout the body. In states of chronic stress and starvation such as AN, there is decreased conversion of T4 to T3, which reduces resting energy expenditure [36], and increased conversion of T4 to the metabolically inactive form of T3, reverse T3. One study found a positive correlation between total T3 levels and percentage of ideal body weight, and a negative correlation between reverse T3 levels and percentage of ideal body weight, in adolescent girls and women with AN [37]. In more severe disease, levels of thyroid stimulating hormone (TSH) from the anterior pituitary and free T4 from the thyroid may fall into the low-normal range due to general suppression of the hypothalamic-pituitary-thyroid axis. This pattern of thyroid function tests is termed non-thyroidal illness. More severe depressive symptoms in AN are also associated with lower free T3 concentrations and with lower free T3/free T4 ratios [38]. If the degree of suppression of T3 and T4 seems out of proportion to the severity of the illness, consider evaluating for causes of central hypothyroidism, such as hypopituitarism.

With weight recovery, total T3 levels increase and reverse T3 levels decrease [37]. However, one study reported that mean serum total T3 levels remained lower in women with AN who had achieved weight recovery than healthy controls [39], and another study reported no significant change in serum TSH or total T4 levels with weight recovery [37]. Mildly low serum T3 levels may persist despite weight restoration if eating disorder psychopathology remains active. Although T3 supplementation has been used to enhance the efficacy of anti-depressant medications in other populations [40], administration of T4 and/or T3 in patients with AN and non-thyroidal illness is not advised as it can increase cardiovascular risk by reversing the low basal metabolic rate and bradycardia that are considered adaptive in the disorder. In addition, T4 and/or T3 supplements have the potential to be abused by patients with AN since they increase basal metabolic rate and can cause weight loss.

## Growth hormone-insulin-like growth factor 1 axis

Pituitary growth hormone (GH) pulsatility and secretion are higher in states of chronic starvation such as AN [41], which may be mediated in part by increased levels of ghrelin, a GH secretagogue [42]. In addition, AN is a state of GH resistance, with lower serum insulin-like growth factor 1 (IGF-1) levels compared to controls despite a higher serum GH level, which further stimulates GH secretion through a negative feedback mechanism. One potential mechanism for GH resistance may be elevated levels of fibroblast growth factor 21 (FGF-21), which inhibits GH’s intracellular effects, including the production of IGF-1 from the liver [43]. Consistent with this, serum FGF-21 levels are higher in women with AN compared to controls and are inversely correlated with serum IGF-1 levels [44]. GH resistance may also be mediated in part by low levels of insulin in chronic undernutrition, which downregulate hepatic GH receptor expression (45). In chronic undernutrition, high levels of GH might be an adaptive response to maintain glucose levels via gluconeogenesis and mobilize fat stores via lipolysis [46], and low IGF-1 levels might preserve energy by decreasing linear bone growth in adolescents. Consistent with this, longitudinal bone growth is negatively affected in adolescent boys with AN [47], although data are discrepant in adolescent girls with the disorder [48, 49].

With weight recovery, alterations in serum GH secretion and IGF-1 levels are reversible [50]. Since changes in the GH/IGF-1 axis are adaptive and reversible with weight restoration, pharmacologic interventions on the GH/IGF-1 axis are not currently recommended in patients with AN. In fact, in a randomized controlled trial, administration of supraphysiologic recombinant human GH (rhGH) by daily SQ injection for 12 weeks in women with AN not only did not increase the serum IGF-1 level, but also resulted in a loss of fat mass [51]. Administration of recombinant human IGF-1 (rhIGF-1) followed by the bisphosphonate, risedronate, increases bone mineral density (BMD) at the lateral spine, but not at other skeletal sites, in women, but not adolescent girls, with AN and low BMD compared to placebo [52, 53]. Recombinant human IGF-1 is not FDA-approved for this indication.

## Hypothalamic hormones antidiuretic hormone and oxytocin

Hyponatremia is common in AN, affecting 16–20% of patients [54, 55]. Causes of hyponatremia in AN include primary polydipsia, hypovolemia due to undernutrition (specifically lack of fluid ingestion) and purging behaviors (vomiting, laxative abuse, diuretic abuse), and a low-protein, high-water diet in which solute intake, and therefore renal solute excretion, is so low that renal water excretion is impaired. Another cause of hyponatremia in AN is the syndrome of inappropriate antidiuretic hormone (SIADH). ADH is a hormone produced in the hypothalamus and secreted by the posterior pituitary that increases water reabsorption from the kidneys. In patients with SIADH, ingestion of water does not adequately suppress ADH, which leads to water retention, increases total body water, and lower the plasma sodium concentration by dilution. Although the prevalence of SIADH in AN is unknown, many psychotropic medications used to treat comorbid anxiety and depression in AN are associated with SIADH [56]. Additional causes of hyponatremia, such as adrenal insufficiency, should be considered if the clinical context is suggestive. All cases of hyponatremia should be evaluated and managed. Severe, and especially acute, hyponatremia can be life-threatening and cause an altered level of consciousness and seizures.

Similar to ADH, oxytocin is synthesized by the hypothalamus, and stored in and released by the posterior pituitary. Patients with AN have lower levels of oxytocin both in the cerebrospinal fluid [57] and the serum [58]. As oxytocin has known anxiolytic and anti-depressant effects, it is hypothesized that low oxytocin levels may contribute to mood disorders in patients with AN [59], but no studies have looked at the effects of oxytocin administration in women with the disorder. Interestingly, postprandial serum oxytocin levels are higher in women with AN than controls [60], which may be adaptive to the stress of food intake in the disorder.

## Gut-derived appetite regulating hormones, ghrelin and peptide YY

Ghrelin, mostly secreted by gastric cells, is orexigenic (an appetite stimulant). Ghrelin is also a GH and ACTH secretagogue [23] and an inhibitor of gonadotropin (i.e., LH and FSH) secretion [61]. Serum levels of ghrelin are higher in women with AN, and lower in women who are constitutionally lean [62], compared to controls. In patients with AN, the lack of a response to increased levels of ghrelin suggests that dysregulation of appetite and feeding, and perhaps ghrelin resistance, may contribute to the pathogenesis of AN. In addition, women with AN have a greater reduction in serum ghrelin levels compared to controls during euglycemic hyperinsulinemic clamp conditions [63], in which plasma insulin concentration is acutely raised and maintained by a continuous insulin infusion while the plasma glucose concentration is held constant by a continuous glucose infusion. These data suggest that an exaggerated suppression of ghrelin by hyperinsulinemia (i.e., as it occurs with eating) may result in increased satiety in patients with AN. Weight recovery results in a reduction in fasting serum ghrelin levels in patients with AN [64].

A couple studies have suggested a potential role of pharmacologic interventions of ghrelin in patients with AN. Exogenous IV ghrelin infused twice a day preprandially for two weeks improved gastrointestinal symptoms including epigastric discomfort and constipation and increased reported feelings of hunger in four out of five patients with AN [65]. In another study, administration of a ghrelin agonist, relamorelin SQ injection daily, for four weeks shortened gastric emptying time but did not result in a significant change in gastrointestinal symptoms or increase in weight in 10 women with AN, although three women randomized to relamorelin discontinued the study medication due to reported feelings of increased hunger [66]. Further studies are needed to better understand the role of exogenous ghrelin and ghrelin agonists in patients with AN.

Peptide YY, secreted by endocrine L cells in the gastrointestinal tract, is anorexigenic (an appetite suppressant). As such, PYY levels should be low in chronic starvation. Yet, fasting plasma PYY levels are paradoxically elevated in women with AN in some studies [28, 67–71], but similar to healthy controls in other studies [72–74]. However, not all studies assessed the biologically active form of PYY, PYY3-36; of those studies that did, data remain discrepant [67, 68, 72]. Interestingly, one study that assessed PYY3-36 demonstrated that plasma PYY3-36 levels increased and remained higher through a test meal in women with AN compared to controls, suggesting that the exaggerated increase in PYY3-36 after eating may result in increased satiety in patients with AN. Consistent with these data, measures of dietary restraint are positively correlated with plasma PYY3-36 levels in women with AN [75]. Further studies are needed to elucidate the role of anorexigenic hormones in AN.

## Glucose homeostasis

Symptomatic hypoglycemia (plasma glucose < 55 mg/dL with symptoms consistent with hypoglycemia) is an uncommon manifestation of anorexia nervosa. It is more often present in more severe forms of the disease in patients with BMI < 15 kg/m^2^, and may be exacerbated by a robust insulin response during refeeding [76]. Hypoglycemia portends a poor prognosis in AN, as it implies that hepatic glycogen reserves and substrates for hepatic gluconeogenesis are depleted [77]. Chronic starvation may also result in hypoglycemic unawareness, in which patients may not mount the typical symptoms associated with hypoglycemia [78]. Hypoglycemia has been speculated to contribute to the high rate of sudden death in AN, and thus should be evaluated and managed expeditiously.

Eating disorders among patients with type 1 diabetes may manifest as the misuse of diabetes medications, i.e., insulin [79]. Patients with type 1 diabetes mellitus may intentionally reduce or omit doses of insulin to lose weight. Omitting insulin results in a catabolic state because it reduces the amount of glucose that can enter cells; instead, proteins and fats are broken down for energy and glucose is excreted in the urine. Although the reduction or omission of insulin in patients with type 1 diabetes mellitus and eating disorders is associated with very high morbidity and mortality, evidence-based guidance on its treatment is needed, as standard eating disorder treatment models are not effective [79].

## Body composition, adipokines, and myokines

Adolescents and adults with AN have both less fat mass and lean mass than controls [80–82]. It is important to note that although patients with AN have an intense fear of becoming fat, loss of both fat and muscle mass is an unavoidable consequence of weight loss. Studies have shown that adolescent boys with AN have relatively less loss of fat mass in the trunk than the extremities [83], and that with weight regain, both adolescent boys and women regain relatively more fat in the trunk than the extremities [32, 82]. These changes in body composition with weight loss and regain may be due to characteristic hormonal abnormalities in AN that tend to preserve trunk fat, including hypercortisolemia and hypogonadotropic hypogonadism.

Adipokines and myokines are hormones produced by adipose tissue and muscle tissue, respectively. In addition to playing a role in functional hypogonadotropic hypogonadism in AN, deficits in the anorexigenic adipokine, leptin, have been hypothesized to promote starvation-induced hyperactivity in animals [84, 85], which is interesting to note as compulsive exercise is common in patients with AN. Serum leptin levels rise with weight recovery; women with AN who are weight recovered may actually have higher serum leptin levels than controls [86]. Whether these relatively higher leptin levels play a role in difficulty with weight gain, or disease relapse and recurrent weight loss, is unknown. Regardless, recombinant human leptin administration is not advised in women with AN. Although recombinant human leptin administration restored ovulatory menstrual cycles in three out of eight normal-weight women with hypothalamic amenorrhea due to causes other than AN, it also decreased fat mass [87]. Serum levels of myostatin, a myokine that acts as an inhibitor of muscle growth, are lower in women with AN compared to controls [88]. Whether this is a mechanism to prevent even further muscle loss in the setting of weight loss is unknown.

## Bone marrow adipose tissue

In contrast to other adipose depots, bone marrow adipose tissue, assessed non-invasively by proton magnetic resonance spectroscopy (^1^H-MRS), paradoxically increases in the early stages of starvation and is higher in women with AN compared to controls [89]. The reason for this is unknown, but may be mediated in part by higher serum levels of preadipocyte factor 1 (Pref-1). Pref-1 is a negative regulator of osteoblast and adipocyte differentiation and is associated with higher bone marrow adipose tissue content in women with AN [90]. Consistent with this, higher bone marrow adipose tissue content is associated with lower BMD in older adolescent girls and women with AN [89, 90]. In later stages of chronic starvation, such as severe AN, where protein wasting can lead to death, bone marrow adipose tissue content is reduced as it is mobilized and metabolized as a final energy repository in a process called serous atrophy of bone marrow [91].

## Skeletal integrity

Low BMD and increased fracture risk are common and severe medical complications in patients with AN. Adolescence is a period of high bone turnover, with bone formation being greater than bone resorption [92] in order to achieve peak bone mass. In contrast, in adolescent girls with AN, both bone formation and bone resorption are lower compared to controls [93], indicative of reduced bone turnover and often resulting in failure to reach peak bone mass. In women with AN, bone formation is lower and bone resorption is higher than in controls [94], resulting in mean loss of BMD by dual-energy x-ray absorptiometry (DXA) of approximately 2.4% at the hip and 2.6% at the spine annually [95]. For both adolescents and adults with AN, decrements in BMD by DXA may be detected within 12 months of active disease [96]. Given the failure to accrue bone mass in adolescents, and bone loss in adults, with AN, low BMD is a common comorbidity in the disorder. Among pre-menopausal women, low BMD (defined as a BMD Z-score < − 2 at the spine, hip, or radius) is present in 39% of those with AN, and 24% of those with atypical anorexia nervosa (AAN) [81]. The prevalence of low BMD is similar among men with AN and AAN [97]. Studies have also shown that adolescent girls and women with AN have impaired bone microarchitecture and estimated strength at the lumbar spine and tibia and radius as assessed by quantitative computed tomography (QCT) and high resolution peripheral quantitative computed (HR-pQCT), respectively [98, 99]. Fracture risk is increased in women with AN across all ages and at all skeletal sites, whereas increased fracture risk in men with AN is seen after age 40 years [100]. A cross-sectional study in adolescent girls with active AN and an eight-year longitudinal study in adults with AN (regardless of current disease status) did not find an association between BMD by DXA and prevalent or incident fractures, respectively [101, 102]. This may be because (1) the study populations were heterogeneous in terms of duration of disease and disease status, (2) known relationships between BMD and fracture risk are derived from a different population (i.e., post-menopausal women), (3) DXA may not be as accurate at the extremes of the weight spectrum, and (4) DXA does not capture detrimental changes to bone microarchitecture and bone matrix properties that are important determinants of bone strength.

Risk factors for low BMD in patients with AN include onset of disease during adolescence, lower BMI, longer duration of illness, hypogonadotropic hypogonadism, low muscle mass, calcium intake less than 600 mg/day, and lower serum 25OH vitamin D levels [95, 97, 103, 104]. Low serum estradiol and high serum cortisol levels, which stimulate bone resorption and inhibit bone formation, are associated with low BMD in women with AN [27, 105, 106]. Growth hormone resistance with low serum IGF-1 levels characteristic of AN are also associated with low BMD [107] as GH and IGF-1 stimulate osteoblast (cells responsible for new bone formation), and inhibit osteoclast (cells responsible for bone resorption), differentiation [108]. Oxytocin is also thought to promote osteoblastic over osteoclastic activity, and lower serum overnight oxytocin levels in women with AN are associated with lower BMD [58]. Studies have reported an inverse association between bone marrow adipose tissue and BMD in women with AN [89, 90].

No formal guidelines exist for BMD screening by DXA in patients with AN. However, given the prevalence of low BMD and increased risk of fractures in this population, BMD screening should be considered in patients with AN, particularly those with multiple risk factors for low BMD. Specifically, the Endocrine Society Clinical Practice Guidelines for functional hypothalamic amenorrhea recommend baseline BMD measurement by DXA in any adolescent girl or women with six or more months of functional hypothalamic amenorrhea or sooner if there is a history or suspicion of severe nutritional deficiency and/or skeletal fragility [109]. As guidelines do not exist regarding the optimal time to reassess BMD after an initial screening DXA in patients with AN, the decision should be individualized according to factors such as the clinical context, the BMD at initial screening, and insurance coverage; an interval of 1–2 years could be considered. A diagnosis of low BMD may motivate patients with active AN to seek eating disorder treatment as weight recovery and restoration of menses can result in significant improvements in BMD. Indeed, weight restoration and resumption of menstrual periods are the cornerstone of therapy for low BMD as no medications are currently FDA-approved for the treatment of low BMD in AN. Women with AN who gain weight and resume menses have an annual mean increase in spine BMD of 3.1%, whereas those who do not recover menstrual function regardless of whether weight is regained have an annual mean decline in spine BMD of 2.4% [95]. It is important to be realistic with patients that the deleterious effects of AN on BMD may not be completely reversible with weight restoration and restoration of menses, particularly for those patients who did not achieve their peak bone mass during adolescence.

Other diet and lifestyle factors are also important to consider in the treatment of low BMD in patients with AN. Although low calcium and vitamin D intake have been associated with low BMD in the disorder, adequate calcium and vitamin D intake alone is not adequate to prevent low BMD in patients with AN [55]. In addition, supplemental calcium and vitamin D intake is higher in adolescent girls and women with AN compared to healthy controls [110]. Exercise programs must be individualized in patients with AN as exercise that results in a state of energy deficiency has deleterious effects on bone [111].

Oral contraceptive pills (OCPs) are not recommended as a treatment for low BMD as they do not correct the nutritional and endocrine abnormalities that cause functional hypogonadotropic hypogonadism and low BMD [112, 113]. In addition, oral estrogen further suppresses hepatic IGF-1 secretion [114], which may worsen BMD. Although one study reported that women with AN taking OCPs had higher BMD than those who were not taking OCPs, this was a cross-sectional study with potential confounding variables, not a randomized controlled trial [115]. In contrast to oral estrogen, transdermal estradiol may be beneficial to BMD in adolescent girls and women with AN. In adolescent girls with AN, administration of transdermal estradiol (100mcg patched applied twice weekly) with oral medroxyprogesterone 2.5 mg daily for 10 days each month increased, but did not normalize, spine BMD compared to placebo over 18 months [116]. In women with AN, an open-label weekly transdermal estradiol (0.045 mg/day) and levonorgestrel (0.015 mg/day) patch increased spine BMD over 6 months [117]. It is important to note that the doses of transdermal estradiol used in these studies do not provide contraceptive benefit and that patients must be informed about this. The Endocrine Society Clinical Practice Guidelines suggest that clinicians may consider short-term use of transdermal estradiol with cyclic oral progestin in adolescent girls and women with hypothalamic amenorrhea if menstrual function has not resumed after 6–12 months of nutritional, psychological, and exercise-related interventions in those with low BMD, regardless of whether the interventions were successful in weight recovery [109]. This is because bone metabolism may be compromised even after just 6–12 months of amenorrhea. Although testosterone has been shown to increase BMD in older men with osteoporosis and younger men with hypogonadism [118, 119], a randomized controlled trial of testosterone replacement in adolescent boys and men with AN has not been performed.

Bisphosphonates, denosumab, and teriparatide, which are all approved for the treatment of osteoporosis in postmenopausal women, have been studied in women with AN. However, no therapies are currently FDA-approved for the treatment of low BMD in patients with AN. Risedronate, a bisphosphonate that suppresses bone resorption, increases spine BMD by 3.2% and hip BMD by 1.9% over 12 months in women with AN [120]. However, bisphosphonates have not been shown to increase BMD in adolescent girls with AN [121], perhaps because bone resorption is already low in this population. In addition to not being FDA-approved for the treatment of low BMD in pre-menopausal women with AN, bisphosphonates have a long half-life and are known to cross the placenta, which are considerations to their use in women of child-bearing potential. However, case reports of bisphosphonates in pregnant women have been reassuring with respect to effects on the fetus [122]. Studies have shown that rhIGF-1, which addresses the relative IGF-1 deficiency in the disorder, followed by risedronate increases BMD at the lateral spine, but not at other skeletal sites, in women with AN and low BMD compared to placebo [52]. Recombinant human IGF-1 is not FDA-approved for this indication. Denosumab, another antiresorptive therapy, increases spine BMD by 5.5% over 12 months in women with AN, but is not FDA-approved for the treatment of low BMD in this population [52]. Teriparatide, a bone anabolic agent, increases spine BMD by 6.0% in women with AN after 6 months compared to placebo [123]. However, this drug is also not FDA-approved for the treatment of low BMD in patients with AN.

## Conclusions

An important component in the treatment of AN is the evaluation and management of its endocrine complications, including functional hypogonadotropic hypogonadism and increased fracture risk. The body’s adaptive response to chronic starvation results in many endocrine abnormalities, most of which are reversible upon weight restoration. For example, weight restoration results in resumption of menstrual cycles in most, but not all, women with AN. Weight recovery and restoration of menstrual periods are critical factors to address when managing low BMD. However, low BMD may not be completely reversible with weight restoration and restoration of menses, particularly for those patients who did not achieve their peak bone mass during adolescence. A multidisciplinary team with experience in treating patients with AN is critical to improving endocrine outcomes in patients with this disorder, including in women with AN who are interested in fertility. Much less is understood about endocrine complications in men, as well as sexual and gender minorities, with AN. Future research in endocrine complications in AN must improve representation of these historically underrepresented groups.

## Data Availability

Not applicable.

## Abbreviations

AN: Anorexia nervosa
AAN: Atypical anorexia nervosa
DSM-5: Diagnostic and Stastical Manual of Mental Disorders, 5th edition
GnRH: Gonadotropin-releasing hormone
CRH: Corticotropin-releasing hormone
DHEAS: Dehydroepiandrosterone sulfate
T4: Thyroxine
T3: Triiodothyronine
TSH: Thyroid stimulating hormone
GH: Growth hormone
IGF-1: Insulin-like growth factor 1
FGF-21: Fibroblast growth factor 21
rhGH: Recombinant human GH
rhIGF-1: Recombinant human IGF-1
BMD: Bone mineral density
SIADH: Syndrome of inappropriate antidiuretic hormone
^1^H-MRS: Proton magnetic resonance spectroscopy
Pref-1: Preadipocyte factor 1
DXA: Dual-energy x-ray absorptiometry
QCT: Quantitative computed tomography
HR-pQCT: High resolution peripheral quantitative computed tomography
OCP: Oral contraceptive pill
